# The validation of a serious game for teaching ultrasound skills

**DOI:** 10.1186/s13089-022-00280-8

**Published:** 2022-07-23

**Authors:** T. J. Olgers, J. M. van Os, H. R. Bouma, J. C. ter Maaten

**Affiliations:** Department Internal Medicine, University of Groningen, University Medical Center Groningen, 9700 RB Groningen, The Netherlands

**Keywords:** POCUS, Serious games, Ultrasound education, Ultrasound training, Internal medicine

## Abstract

**Background:**

Point-of-care ultrasound (POCUS) is an important bedside diagnostic tool and is being taught in several specialties. However, mastering the required psychomotor skills takes time and learning curves are different between students. Especially learning to make the right probe movements with the corresponding changes of the ultrasound image on screen, and integrating it into a 3D mental model takes time. This precious bedside-time of trainers and physicians may be reduced using other learning methods for mastering the psychomotor skills, for example the use of serious games. Such a game is under development but it needs to be validated before widespread use can be advised. In this article we describe the development and the first three steps in the validation of a serious game for ultrasound skills.

**Results:**

We have included 18 ultrasound experts and 24 ultrasound novices who played the serious game ‘Underwater” and provided feedback. They concluded that “underwater” is fun to play and that movement of the 3D-printed probe resembled real ultrasound probe movements. Participants highly valued the potential of the game for training eye–hand coordination and stability of probe handling, two very important skills in performing ultrasound in real practice. Although we compared several in-game parameters such as distance and speed, no difference was observed between novices and experts. This means that content- and face validity of the serious game is demonstrated but optimal parameters to measure differences between novices and experts still have to be determined.

**Conclusions:**

Our study shows solid content- and face validity of the serious game “UnderWater” for training ultrasound skills, although construct validity could not be demonstrated yet. The game is appreciated as a promising serious game for training eye–hand coordination and learning ultrasound, which may reduce expensive bed-side teaching.

**Supplementary Information:**

The online version contains supplementary material available at 10.1186/s13089-022-00280-8.

## Background

Ultrasound is an important diagnostic imaging tool traditionally performed by radiologists. Point-of-care ultrasound (POCUS) is being performed by several clinical specialties including emergency medicine, critical care and internal medicine [1–3]. POCUS is the bedside application of ultrasound, usually performed by the treating physician with a specific and often binary question. In this way, the physician is not dependent on the radiologist to perform the examination in a timely and clinically relevant manner. However, correctly interpreting the images—as POCUS is highly user-dependent—requires the acquisition of very specific knowledge and skills Users need to master probe handling, optimizing images, and constructing a mental 3D model of the specific organ. Mastering these psychomotor skills takes time and it may not be cost-effective to learn this at the bedside.

Serious gaming is a new training modality that can complement traditional bedside teaching. The added value has already been demonstrated in surgery by the game “Underground” that improves laparoscopic skills [4]. Yet, assessing the validity of a serious game is essential before it can be implemented into medical education. The validity of a serious game for a technical skill means that playing the game will actually improve the specific technical skill in real life. Validation is the process of collecting and interpreting validity evidence. So far, only a few studies have described the validation of serious games for learning technical skills in medicine and most of these games are not fully validated [5]. Several frameworks have been used for the validation of serious games, which all have two key elements: first, to show evidence about the construct of the game itself and, second, evidence that playing the serious game improves the technical skills in real life. The framework described by Graafland and Warmelink distinguishes five different phases of validity [6, 7]. In short, the first three phases consist of content validity, face validity, and construct validity. Content validity concerns the content (specifications) of the game itself: is it legitimate? Are the objectives of the game clear and correct? Face validity means providing evidence that the game appears similar to the construct it attempts to represent. In our game, playing the game and handling the controller should resemble handling a real ultrasound probe. Construct validity indicates that the game actually measures what it intends to measure. Is the game able to measure differences between groups, for example experts and novices? These first three stages of validation describe evidence about the game itself. The last two phases of validation consist of evidence that playing the game will actually improve the technical skills in real life: the correlation or prediction of the performance scores in the game and the performance in real life (concurrent and predictive validity, respectively). To our best knowledge, no serious game is currently available to learn ultrasound skills, but such a game is currently being developed in the Netherlands [8]. In this article we will describe the first three steps in the validation of this game.

## Methods

### Game development

**Fig. 1:**
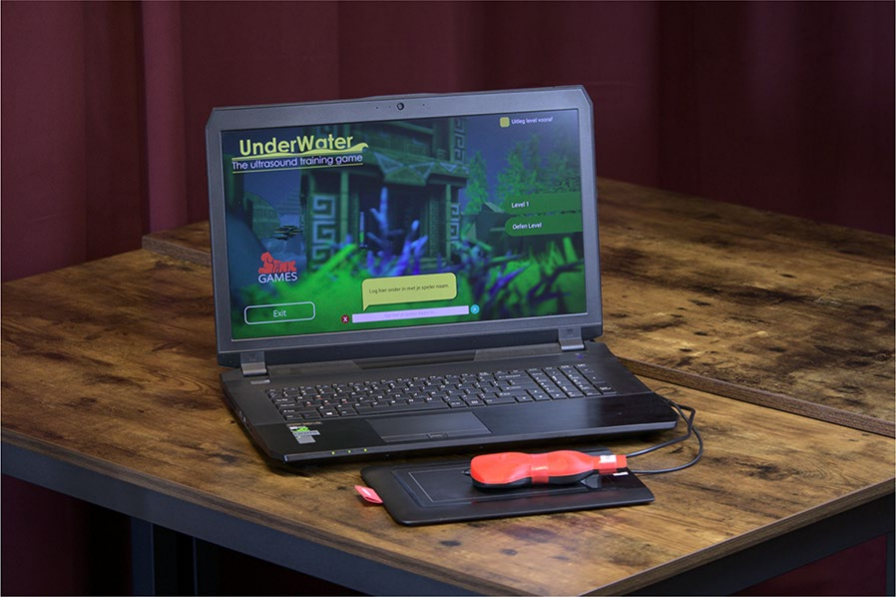
Game setup

In 2017, game developers contacted our ultrasound research group for a demonstration of the game “UnderWater”. The setup for this game is a stylus pencil within a 3D printed ultrasound probe, a touchpad and a laptop with the game software “UnderWater” (Fig. 1). The probe has a similar shape and cord as a real ultrasound probe. The player can manipulate the game probe in all directions to move the in-game pointer in which the probe movements resemble the real-life ultrasound probe movements. The surface of the tablet resembles the total virtual playground (no lifting of probe needed). The purpose of the game is to collect coins in an underwater world. The coins are hidden between rock buildings and/or aquatic plants on the sea floor. If a coin is located, the player needs to move and keep still the pointer exactly on the coin for 1 s to collect it. The player must use several, and sometimes combined movements to be able to collect the coins. These necessary movements resemble real-life probe movements, for example tilting, rocking and sliding. Using these movements, coins hidden under objects are made visible and collectable. A level consists of ten different coins in which the next coin will only become visible if the previous one is collected.

During several meetings after the first demonstration of the game, we discussed the content of the game and the resemblance with real life probe handling. In 2018, we tested the game during a few ultrasound courses with both ultrasound novice participants and expert ultrasound instructors who played the game and provided feedback about the game itself (content validity) and the resemblance with performing ultrasound in real-life (face validity). In the first versions of the game, scores were time-based. However, participants had trouble orienting themselves in the game field and finding coins. Consequently, final scores reflected the ability (or inability) to find the coins in general, but not specifically probe movements as a technical skill. In 2019, we have tested the game at a large internal medicine assembly in the Netherlands (annual Dutch Internal Medicine Assembly 2019). Participants of the assembly were able to play the game and compare their scores in a local competition. We have asked them to provide us with feedback about their experience with the game.

**Fig. 2:**
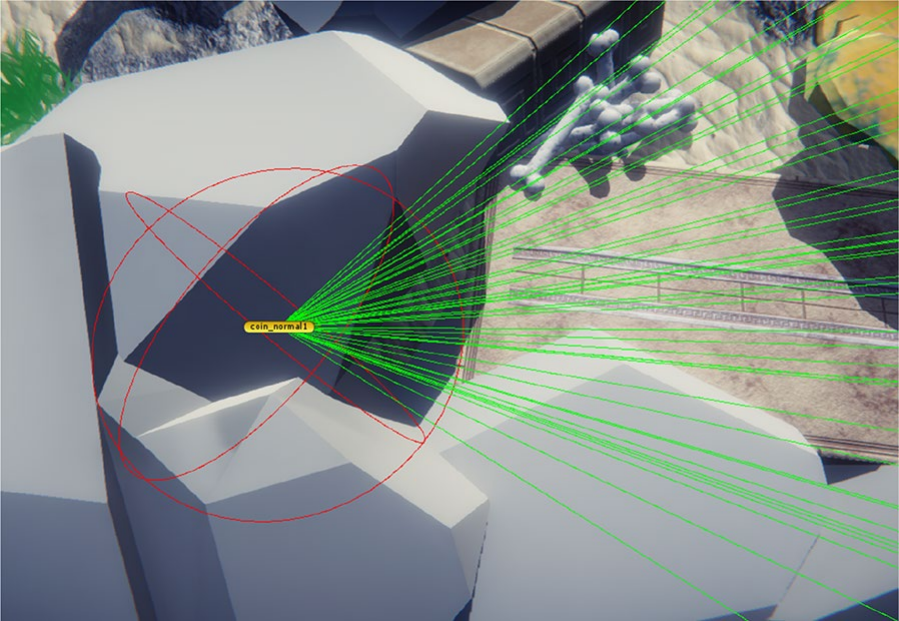
Time-bubble and ideal path

In 2020,”UnderWater” was adjusted following the results of the abovementioned testing rounds. First, the developers added an in-game help tool: a sonographic wave which is sent across the sea floor intermittently. The red reflection of this wave hints at the direction of the hidden coin. By pressing the space bar during the game, this hint can be repeated as many times as needed. This addition improves the ability of players to localize the coin in the total playfield. Second, specific software was developed in conjunction with the University of Groningen which enabled us to measure more exact probe movements and to relate them to an ideal path configured by the software. In addition, the game software is able to measure time and movement of the probe when the coin is visible for the participant, but not yet collected, by creating an invisible bubble around the coin. If the cursor is within the bubble, the scoring system is activated (Fig. 2). In this way, total scores may better reflect the exact movements of the probe for collecting the coin and not the search for the coin within the whole level. Distance is an arbitrary value of the total distance moved in the game field by the cursor. The value itself has no specific units but should be used to compare players in which a higher value indicates a longer distance. The same is true for speed. These changes resulted in the current 2021 version which was used for the study described in this article.

### Participants

A convenience sample of 42 participants was taken based on previous serious games validation literature [9]. Participants were eligible if they were working as an internist, emergency physician or emergency department (ED) nurse in our university hospital. The majority of participants of this study (residents and staff) are comparable to that of ultrasound courses for learning ultrasound in real-life. Participants were included if they fulfilled the criteria for expert or novice. Experts were defined as those who had practiced ultrasound on a regular basis in the past year and who had performed at least 150 ultrasound exams in total. Novices were defined as not actively performing ultrasound in real life, having made less than 10 ultrasound exams in total, and not having received significant ultrasound training (< 4 h of ultrasound course sessions). Assessment with the game was done in private rooms with the mobile underwater game setup. Participants were not able to see each other play the game. They all received the same instruction and they were able to play one training level with four clearly visible coins. All participants provided informed consent.

### Questionnaire

A paper-based questionnaire was constructed with specific questions about previous gaming experience, ultrasound experience, and questions concerning the game itself. The questionnaire for experts also contained questions to assess whether the game resembles performing ultrasound in real life and its potential value as an educational tool. The questionnaire can be downloaded as Additional file 1.

### Statistical analysis

Statistical analysis was performed with IBM SPSS statistics version 23. Data are presented as absolute numbers, percentages and means. Differences between groups were compared using a Mann-Whitney *U* test.

## Results

In total, 18 Experts and 24 novices were included, of which the demographics are shown in Table 1.

**Table 1:** Demographics of participants

*N* = 42	Expert/Novice *N* (%)	Male/Female *N* (%)
Specialty		
Emergency nurse *N* = 17 (40%)	1 (5)/16 (95)	3 (18)/14 (82)
Emergency physician *N* = 10 (24%)	10 (100)/ 0 (0)	4 (40)/6 (60)
Internist *N* = 10 (24%)	4 (21)/6 (79)	6 (60)/4 (40)
Resident *N* = 3 (7%)	2 (67)/1 (33)	0 (0)/3 (100)
Rheumatologist *N* = 2 (5%)	1 (50/1 (50)	0 (0)/2 (100)
Regular gamer		
Yes 11 (26%)	7 (64)/4 (36)	5 (45)/6 (55)
No 31 (74%)	11 (35)/20 (65)	9 (29)/22 (71)

*N *number of participants

Participants played the same level of the game, representing the same hiding places and number of coins. Due to technical failure of equipment, three participants were unable to finish the game and one participant stopped due to inability to collect the fifth coin. All participants completed the questionnaire.

### Content validity

In general, participants were positive about the serious game (Table 2).

**Table 2:** Content validity of the serious game “UnderWater”

N = 42	Very good	Good	Bad	Very bad	No opinion
Useful in training eye–hand coordination	17 (40.5)	21 (50.0)	3 (7.1)	0	1 (2.4)
Useful in training stability of probe	16 (38.1)	21 (50.0)	4 (9.5)	0	1 (2.4)
UnderWater game fun to play	19 (45.2)	21 (50.0)	2 (4.8)	0	0
UnderWater game challenging	11 (26.2)	21 (50.0)	8 (19.0)	2 (4.8)	0
Did you feel skillful	3 (7.1)	25 (59.5)	8 (19.0)	6 (14.3)	0

Data displayed as absolute numbers and percentages* N*(%)

The purpose of the game was clear, it was fun to play, and also challenging. The majority felt skillful while playing the game, which suggests that handling the controller is easy to learn. Participants were able to make suggestions for improvement of the game through open questions. One important improvement in the game design could be that the cursor in the game field holds its position if the probe is lifted from the tablet instead of centering it. In addition, the game is probably not challenging enough if played for a longer time period. This may be addressed by adding several levels in the future or other gaming elements instead of collecting coins. Some participants got frustrated if they were not able to collect a coin. The game design may help by adding tips or the possibility to proceed to the next coin without collecting the difficult one.

### Face validity

The majority of participants indicated that the 3D-printed game probe feels realistic compared to a real ultrasound probe and that the movement of the probe resembles the movement in real life practice including the movement of the cursor on the screen (Table 3).

**Table 3:** Face validity of the serious game “UnderWater”

N = 42	Very good	Good	Bad	Very bad	No opinion
Realistic compared to real probe	6 (14.3)	27 (64.3)	5 (11.9)	0	4 (9.5)
Moves such as real probe	5 (11.9)	22 (52.4)	5 (11.9)	1 (2.4)	9 (21.4)
Moves cursor on screen such as real probe	7 (16.7)	17 (40.5)	8 (19.0)	1 (2.4)	9 (21.4)
Ergonomic compared to real probe	6 (14.3)	22 (52.4)	6 (14.3)	0	8 (19.0)
Generally useful for training real ultrasound probe handling	8 (19.0)	28 (66.7)	3 (7.1)	0	2 (4.8)

Data displayed as absolute numbers and percentages* N*(%)

This means that making a specific movement with the probe and thereby moving the cursor in a specific direction, resembles the same way of “looking around” with real ultrasound movements. The game probe is ergonomically comparable with the real probe. Participants highly value the potential of the game for training eye–hand coordination and stability of probe handling, two very important skills in performing ultrasound in real practice. However, following the results of the open questions, the design of the game probe needs improvement: it should be made more realistic. This means increasing its weight compared to a real probe and providing a better and larger contact area of the probe with the tablet. In addition, a direction marker on the probe would be useful to make it resembling real-life probes.

### Construct validity

Several parameters were calculated during the game, including total time played, average number of attempts per coin, time spent in bubble, probe distance, probe speed, loss of contact from tablet, and final score. Only time spent in bubble (cursor is in vicinity of the coin and coin is visible for participant) was slightly lower in the novices group (Table 4) but this difference disappears when adjusting for total playing time. Other parameters were not significantly different.

**Table 4:** Construct validity of the serious game “UnderWater”

	Experts (N = 17)	Novices (N = 21)	*P* value
Time (sec)	601.7	433.6	0.058
Attempts	2.05	2.5	0.070
Time in bubble (sec)	16.8	12.7	*0.048*
Percentage in bubble from total playing time	26.38	26.62	*0.714*
Distance	104.1	71.4	0.081
Speed	1.7	1.53	0.895
Loss of contact (times)	59.1	51.7	0.649
Final score	0.87	0.88	0.953

Data displayed as means. Distance and speed displayed as arbitrary software data for comparison only (not in standard units). Final score is based on calculations of multiple parameters

### Educational tool

We asked the participants what parts of the game, probe or scores they thought were crucial for using the game “UnderWater” as an educational tool. A substantial portion of the participants (40%) had no specific opinion, but for the others most items were crucial or at least useful. Most participants valued this game as an educational tool, especially for evaluating and training probe-handling (Table 5).

**Table 5:** “UnderWater” as an educational tool

N = 42 (%)	Yes	No	No opinion
Necessary to use serious game in ultrasound learning trajectory	13 (31.0)	11 (26.2)	18 (42.9)
UnderWater game useful for judging probe handling	26 (61.9)	4 (9.5)	12 (28.6)
UnderWater game useful for training probe handling	32 (76.2)	1 (2.4)	9 (21.4)
UnderWater game potentially cost-effective for training probe skills	23 (54.8)	2 (4.8)	17 (40.5)

Data displayed as absolute numbers and percentages ()

They indicate this may be a cost-effective alternative to traditional bedside teaching. For example, the use of obstacles, searching for coins to challenge probe movements, and calculating scores compared to the ideal path were most valued (Table 6).

**Table 6:** Crucial elements of the serious game “UnderWater” according to experts

*N* = 17 (%)	Crucial	Useful	Not necessary
Attempts on coin	4 (23.5)	11 (64.7)	2 (11.8)
Distance of probe	2 (11.8)	12 (70.6)	3 (17.6)
Speed of probe	1 (5.9)	13 (76.5)	3 (17.6)
Total time played	2 (11.8)	13 (76.5)	2 (11.8)
Lifting of probe	2 (11.8)	9 (52.9)	6 (35.3)
Ideal path	5 (29.4)	11 (64.7)	1 (5.9)
Use of obstacles	9 (52.9)	8 (47.1)	0
Finding coins	4 (23.5)	12 (70.6)	1 (5.9)
Shape of probe	4 (23.5)	13 (76.5)	0

*Data displayed as absolute numbers and percentages N(%)*

Using probe speed or probe distance was considered least important as a scoring item and lifting of the probe from the tablet. Finally, the experts stated that the game is best used prior to bedside teaching or concurrent with bedside teaching and especially suited for novices. Only one participant felt this game is of little added value.

## Discussion

Our study demonstrates that the serious game “UnderWater” possesses solid content and face validity, the first two steps in the validation of serious games. The third step, construct validity, could not be proven. Participants experienced this game as fun to play and challenging with clear objectives (content validity). The 3D-printed probe resembled the ultrasound probe in real-life in terms of its form. Furthermore, the movement of the game probe, with subsequent changes of the cursor within the game, resembles the effect of probe movement in real-life in three dimensions (face validity). Although some improvements are needed (for example probe adjustments including a larger contact area), participants rate the game as a valuable tool for learning and mastering ultrasound skills. Together, we have demonstrated that the serious game “UnderWater” is a promising additional training tool for mastering ultrasound skills, which is in line with other studies describing a positive effect of serious games to learn technical skills [10].

Unfortunately, we were unable to demonstrate construct validity, meaning the game was unable to measure differences between novices and experts. This might be explained by confounders not accounted for, or the fact that the optimal parameter to differentiate between these two groups is not known yet. In addition, parameters may not be significant due to the relatively small sample size. We hypothesized that total time played may be shorter in novices as experts may take more time to move more precisely to the target. Although the study was not designed to assess time to find coins within a single game level, we attempted to analyze this effect per coin, which also allowed to correct for difficulty of the game level. However, we were not able to detect significant differences between the two groups. Furthermore, we have not measured exact probe movements or how goal-directed the individual subtle movements were, which will be assessed in a follow-up study. Further development and analysis of the scoring system and measurement system is needed. There is no guideline or minimal requirement to conclude whether the first steps of validation of serious games are sufficient, making it a subjective measurement. Other studies have used comparable methods and numbers of participants for validation of other serious games or simulators [11–13]. Apart from that, we have used the framework from Graafland and Warmelink for validation of our game. However, different frameworks for game designing or validation exist [14]. Another commonly used framework for serious game validation is the co.LAB Generic Framework for Collaborative Design of Serious Games. This framework defines 5 main categories: (1) Context and objectives, (2) Game design, (3) Learning design, (4) Mechanics, and (5) Assessment. The most critical category is the ‘mechanics’, defined as ‘patterns of behavior or building blocks of learner interactivity, which may be a single action or a set of interrelated actions that form the essential learning activity that is repeated throughout a game’. Game mechanics have a double objective, resulting in 2 constraints: (1) engaging participants in taking part in the game and (2) ensuring consistency with learning mechanics. In our tested serious game “UnderWater”, this translates into 3D-printed probe movement repetition and collecting coins in which the game controller resembles movements in real practice. Both objectives of game ‘mechanics’ are met in the game “Underwater” as are the main other categories (1) context and objectives, (2) Game design and (3) Learning design. The last category ‘assessment’, how the game and its objectives will be evaluated, is part of the overall design. This may include game assessment by participants, learning assessment within the game itself, or assessments outside the game. For “UnderWater”, the assessment includes measuring time and attempts for coin collection. For subsequent studies, assessment can also consist of comparing two groups performing a real ultrasound, in which one group plays the serious game prior to the ultrasound and compare them with the ultrasound skills of the group not playing the game priorly.

Mastering ultrasound skills, especially eye–hand coordination, takes time and practice. Using a serious game may reduce hands-on practice time and, therefore, be a cost-effective additive, but it is important to emphasize that it will not be a replacement of hands-on practical training. Multiple training modalities may complement each other in mastering technical skills [15]. Another advantage of a serious game is that participants are not dependent on the availability of an experienced ultrasound trainer. The best moment for introducing this serious game in ultrasound learning, according to the participants, is before or concurrent with hands-on sessions, and would be especially useful for novices in ultrasound. However, we believe it might also be challenging and valuable for more experienced sonographers if the difficulty of the game levels can be increased.

## Conclusions

Serious games are a fun add-on to traditional teaching but need to be validated before widespread use can be advocated. This study shows solid content- and face validity of the serious game “UnderWater” for training ultrasound skills, although construct validity could not be demonstrated using the framework from Graafland and Warmelink. “UnderWater” is appreciated as a promising serious game for training eye–hand coordination and learning ultrasound, which may reduce expensive bed-side teaching.

## Supplementary Information

**Additional file 1.** Questionnaire UnderWater.

## Data Availability

The data sets used and/or analyzed during the current study are available from the corresponding author on reasonable request.

## Abbreviations

POCUS: Point-of-care ultrasound
ED: Emergency department

## References

[1] Whitson MR, Mayo PH (2016). Ultrasonography in the emergency department. Crit Care.

[2] Leidi A, Rouyer F, Marti C, Reny JL, Grosgurin O (2020). Point of care ultrasonography from the emergency department to the internal medicine ward: current trends and perspectives. Intern Emerg Med.

[3] Olgers TJ, Azizi N, Blans MJ, Bosch FH, Gans ROB, Ter Maaten JC (2019). Point-of-care ultrasound (PoCUS) for the internist in acute medicine: a uniform curriculum. Neth J Med.

[4] IJgosse W, van Goor H, Rosman C, Luursema JM (2020). Construct validity of a serious game for laparoscopic skills training: validation study. JMIR Serious Games.

[5] Olgers TJ, Bij de Weg AA, Ter Maaten JC (2021). Serious games for improving technical skills in medicine: scoping review. JMIR Serious Games.

[6] Graafland M, Dankbaar M, Mert A (2014). How to systematically assess serious games applied to health care. JMIR Serious Games.

[7] Warmelink H, Valenta M, Van Tol R, Schravenhoff R, De Gloria A, Veltkamp R (2015). Get It right! Introducing a framework for integrating validation in applied game design. Book Games and Learning Alliance.

[8] Website sfinx games. https://www.sfinxgames.com. Accessed 15 July 2022

[9] Jalink MB, Goris J, Heineman E, Pierie JP, ten Cate Hoedemaker HO (2014). Construct and concurrent validity of a Nintendo Wii video game made for training basic laparoscopic skills. Surg Endosc.

[10] Jalink MB, Goris J, Heineman E, Pierie JP, Ten Cate Hoedemaker HO (2015). Face validity of a Wii U video game for training basic laparoscopic skills. Am J Surg.

[11] Stunt JJ, Kerkhoffs GM, van Dijk CN, Tuijthof GJ (2015). Validation of the ArthroS virtual reality simulator for arthroscopic skills. Knee Surg Sports Traumatol Arthrosc.

[12] Bouaicha S, Epprecht S, Jentzsch T, Ernstbrunner L, El Nashar R, Rahm S (2020). Three days of training with a low-fidelity arthroscopy triangulation simulator box improves task performance in a virtual reality high-fidelity virtual knee arthroscopy simulator. Knee Surg Sports Traumatol Arthrosc.

[13] Jalink MB, Heineman E, Pierie JP, ten Cate Hoedemaker HO (2015). The effect of a preoperative warm-up with a custom-made Nintendo video game on the performance of laparoscopic surgeons. Surg Endosc.

[14] Jaccard D, Suppan L, Sanchez E, Huguenin A, Laurent M (2021). The co.LAB generic framework for collaborative design of serious games: development study. JMIR Serious Games.

[15] Rosser JC, Liu X, Jacobs C, Choi KM, Jalink MB, Ten Cate Hoedemaker HO (2017). Impact of super monkey ball and underground video games on basic and advanced laparoscopic skill training. Surg Endosc.

