# Supplementary material for: The validation of a serious game for teaching ultrasound skills
Source: Ultrasound J. 2022 Jul 23;14:29. doi: 10.1186/s13089-022-00280-8 (PMC9308840; doi:10.1186/s13089-022-00280-8)
Supplement: Supplementary file 1 — Additional file 1. Questionnaire UnderWater. [file 13089_2022_280_MOESM1_ESM.docx]

Questionnaire *UnderWater*

Thank you for playing the game *UnderWater*. This game was developed in the context of scientific research on ultrasound skills training. The purpose of this study is to assess the validity of UnderWater. It examines the correlation between test subjects’ performance in this game and the amount of ultrasound and/or videogame experience they might already have. The questions below are intended to provide insight in this correlation. Your data will be analyzed anonymously. The questionnaire consists of three parts. The first part is completed before playing the game, the rest afterwards.

- I consent to the use of my data.
- I have no objection to being contacted by email for additional questions. My email address is: ......................................................................................................

  I agree to participate in this study (cross out what does not apply): Yes / No

# Questionnaire UnderWater Game: Part 1

The first part of the questionnaire examines your experience in making ultrasound scans.

1. What is your role?

- Senior house officer
- Specialty trainee
- Consultant
- Other, namely _________________________________________________

1. How many ultrasound scans have you performed??

- Less than 10
- Less than 150, but more than 10, namely______________
- 150 or more

1. How many years of experience do you have in making ultrasound scans??

- Less than 4 years
- 4 years or more

1. Did your last ultrasound experience take place in the past year??

- Yes
- No
- Does not apply

1. Have you completed a POCUS training?

- Yes, namely ___________________________________________________
- No

1. Have you followed another ultrasound course of at least 4 hours?

- Yes
- No

# Questionnaire UnderWater Game: Part 2

The following questions concern factors that, in addition to your experience with ultrasound scans, may be related to the score obtained during the video game. Among other things, they handle your experience in playing video games. A video game refers to a visual electronic game that is played on a (game) computer or a mobile device. The amount of video game experience is measured on a scale from 1 to 7, as demonstrated below.

| **1** | Never played a video game |
| --- | --- |
| **2** | Very rarely played a video game |
| **3** | About once a month |
| **4** | A few times a month |
| **5** | Once a week |
| **6** | A few times a week |
| **7** | Daily |

1. How much experience have you (had) playing video games in the following periods in your life? (circle the answer)

| Aged 4-12 (primary school) | 1 | 2 | 3 | 4 | 5 | 6 | 7 |
| --- | --- | --- | --- | --- | --- | --- | --- |
| Aged 12-18 (high school) | 1 | 2 | 3 | 4 | 5 | 6 | 7 |
| Student | 1 | 2 | 3 | 4 | 5 | 6 | 7 |
| Senior house officer/specialty trainee | 1 | 2 | 3 | 4 | 5 | 6 | 7 |
| Currently | 1 | 2 | 3 | 4 | 5 | 6 | 7 |

1. With which game controller do you play the most or have you played the most? You may selectmore than one answer.

- Playstation/Xbox controller
- Wii remote
- Touchscreen (smartphone, tablet)
- Mouse and keyboard
- Joystick
- Steering wheel
- VR controller
- Otherwise, namely ______________________________________________
- Does not apply

1. Which video game(s) do you play or have you played the most and on what platform? (you may give multiple answers)
   1. ______________________________________________________________
   2. ______________________________________________________________
   3. ______________________________________________________________
2. What is your gender?

- Male
- Female

1. What is your age?

______ years

1. What is your dominant hand?

- Left
- Right
- Ambidextrous

1. Has your vision been corrected with glasses, lenses or surgery?

- Yes
- No

1. Do you play a musical instrument?

- Yes
- In the past, namely ____________________ years ago
- No (continue to question 11)

1. How long are you / have you been actively playing a musical instrument?
   ___________________________________________________________________
2. Which musical instrument do / did you play?

___________________________________________________________________

1. Do you practice a sport?

- Yes
- In the past, namely ____________________ years ago
- No (continue to question 14)

1. Which sports do / did you play?

___________________________________________________________________

1. How long have you been playing this / these sport(s)?

___________________________________________________________________

1. How do you rate your spatial awareness? (circle the answer)

++ + - --

(good) (bad)

# Questionnaire UnderWater Game: Part 3

The following questions are to evaluate UnderWater as a serious game.

1. Circle the answer. The game ultrasound probe
   1. looks realistic compared to the real instrument

++ + - --

(good) (bad

- 1. moves realistically in the physical world compared to the real instrument

++ + - --

(good) (bad)

- 1. displays realistic cursor movement on the monitor when manipulated

++ + - --

(good) (bad)

- 1. is ergonomically realistic compared to the real instrument

++ + - --

(good) (bad)

1. Circle the answer. How useful is UnderWater as a teaching tool
   1. for training hand-eye coordination?

++ + - --

(good) (bad)

- 1. for training to keep the ultrasound probe stable?

++ + - --

(good) (bad)

- 1. in general?

++ + - --

(good) (bad)

1. It is necessary for residency trainees to complete POCUS training before using ultrasound on patients

- Yes
- No
- No opinion

1. It is necessary for specialty trainees to train with serious games

- Yes
- No
- No opinion

1. UnderWater is a useful tool to assess ultrasound probe manipulation

- Yes
- No
- No opinion

1. UnderWater is a useful tool to train ultrasound probe manipulation

- Yes
- No
- No opinion

1. UnderWater is potentially a cost effective teaching tool to train ultrasound probe manipulation

- Yes
- No
- No opinion

1. Circle the answer. To what extent did you
   1. enjoy playing the game?

++ + - --

(very much) (not at all)

- 1. find playing the game challenging?

++ + - --

(easy) (difficult)

- 1. feel agile whilst playing the game?

++ + - --

(very much) (not at all)

1. Do you have any suggestions for improving the game?

____________________________________________________________________________________________________________________________________________________________________________________________________________________________________________________________________________________________________________________________________________________________________________________________________________________________________________________________________________________________________________________________________________________________________________________________________________________________________________________________________________________________________________________________________________________________________________________________________________________________________________________________________________________________________________

Questionnaire *UnderWater*

Content validity

The following questions are to evaluate UnderWater as a teaching tool.

1. The physical game ultrasound probe is for learning ultrasound probe manipulation

- Essential
- Useful, but not essential
- Not necessary

1. The search for the gold coins is for learning ultrasound probe manipulation

- Essential
- Useful, but not essential
- Not necessary

1. The obstacles to bypass in the game are for learning ultrasound probe manipulation

- Essential
- Useful, but not essential
- Not necessary

The following questions concern parameters that are taken into account when calculating the score in the game.

1. The computer has calculated the **ideal angle** for picking up each coin. The score obtained takes into account to which extent the movement of the player corresponds to this. To what extent is this parameter “Essential”; “Useful, but not essential”; or “Not necessary” to learn how to operate an ultrasound probe?

- Essential
- Useful, but not essential
- Not necessary

1. To calculate the score, the **number of times the probe is lifted from the touchpad** is taken into account. To what extent is this parameter “Essential”; “Useful, but not essential”; or “Not necessary” to learn how to operate an ultrasound probe?

- Essential
- Useful, but not essential
- Not necessary

1. The **total playing time** is taken into account to calculate the score. To what extent is this parameter “Essential”; “Useful, but not essential”; or “Not necessary” to learn how to operate an ultrasound probe?

- Essential
- Useful, but not essential
- Not necessary

1. **Speed** is taken into account to calculate the score. To what extent is this parameter “Essential”; “Useful, but not essential”; or “Not necessary” to learn how to operate an ultrasound probe?

- Essential
- Useful, but not essential
- Not necessary

1. The **average distance traveled by the probe** is taken into account to calculate the score. To what extent is this parameter “Essential”; “Useful, but not essential”; or “Not necessary” to learn how to operate an ultrasound probe?

- Essential
- Useful, but not essential
- Not necessary

1. To calculate the score, the **number of attempts to pick up the coin** is taken into account. To what extent is this parameter “Essential”; “Useful, but not essential”; or “Not necessary” to learn how to operate an ultrasound probe?

- Essential
- Useful, but not essential
- Not necessary

1. Do you have any suggestions for improving the game?

____________________________________________________________________________________________________________________________________________________________________________________________________________________________________________________________________________________________________________________________________________________________________________________________________________________________________________________________________________________________________________________________________________________________________________________________________________________________________________________________________________________________________________________________________________________________________________________________________________________________________________________________________________________________________________

1. At what point in the learning path would you use the game UnderWater? (multiple options possible)

- Before practical education
- Simultaneously with practical training for beginners
- Simultaneously with practical training for advanced students
- Otherwise, namely __________________________
